# Prognostic indicators of survival and survival prediction model following extracorporeal cardiopulmonary resuscitation in patients with sudden refractory cardiac arrest

**DOI:** 10.1186/s13613-017-0309-y

**Published:** 2017-08-30

**Authors:** Sung Woo Lee, Kap Su Han, Jong Su Park, Ji Sung Lee, Su Jin Kim

**Affiliations:** 1 Department of Emergency Medicine, College of Medicine, Korea University, Inchon-ro 73, Seongbuk-gu, Seoul 02841 Republic of Korea; 2 Clinical Research Center, Asan Medical Center, 88 Olympic-ro 43-gil, Songpa-gu, Seoul 05505 Republic of Korea

**Keywords:** Cardiac arrest, Extracorporeal life support, Cardiopulmonary resuscitation, Emergency department, Survival, Prediction

## Abstract

**Background:**

Extracorporeal cardiopulmonary resuscitation (ECPR) has been considered in selected candidates with potentially reversible causes during a limited period. Candidate selection and the identification of predictable conditions are important factors in determining outcomes during CPR in the emergency department (ED). The objective of this study was to determine the key indicators and develop a prediction model for survival to hospital discharge in patients with sudden cardiac arrest who received ECPR.

**Methods:**

This retrospective analysis was based on a prospective cohort, which included data on CPR with ECPR-related variables. Patients with sudden cardiac arrest who received ECPR at the ED from May 2006 to June 2016 were included. The primary outcome was survival to discharge. Prognostic indicators and the prediction model were analyzed using logistic regression.

**Results:**

Out of 111 ECPR patients, there were 18.9% survivors. Survivors showed younger age, shorter CPR duration (*p* < 0.05) and had tendencies of higher rate of initial shockable rhythm *(p* = 0.055) and higher rate of any ROSC event before ECPR (*p* = 0.066) than non-survivors. Eighty-one percent of survivors showed favorable neurologic outcome at discharge. In univariate analysis, the following factors were associated with survival: no preexisting comorbidities, initial serum hemoglobin level ≥14 g/dL, and mean arterial pressure ≥60 mmHg after ECPR. Based on multivariate logistic regression, predictors for survival in ECPR were as follows: age ≤56 years, no asystole as the initial arrest rhythm, CPR duration of ≤55 min, and any return of spontaneous circulation (ROSC) event before ECPR. The prediction scoring model for survival had a c-statistic of 0.875.

**Conclusions:**

With careful consideration of differences in the inclusion criteria, the prognostic indicators and prediction scoring model for survival in our study may be helpful in the rapid decision-making process for ECPR implementation during CPR in the ED.

## Background

According to recent guidelines, extracorporeal cardiopulmonary resuscitation (ECPR) may be considered for patients with cardiac arrest of potentially reversible etiology during a limited period of mechanical support in settings where it can be rapidly implemented [1, 2]. ECPR, as an alternative resuscitative method for patients with refractory cardiac arrest despite advanced cardiac life support, has been undertaken in the emergency department (ED), intensive care unit, or catheterization room [3]. Survival to discharge rates of ECPR have been reported to be 4–36% for adult out-of-hospital cardiac arrest (OHCA) and 34–46% for adult in-hospital cardiac arrest (IHCA) [4–7]; survival to discharge rates following conventional CPR (CCPR) have been estimated at 10–20% for cardiac arrest [8–11]. Although ECPR was associated with a better outcome than CCPR, the wide range of outcomes is likely to result from the use of different participant selection criteria, protocols, and strategies, according to relevant regional emergency medical services and hospital response systems [12–15].

OHCA cases differ from cardiac arrests occurring during hospitalization in terms of the characteristics of patients, common etiologies of arrest, preexisting disease, low-flow time, and bystander CPR quality [16, 17]. The magnitude of the ECPR effect is more dependent on patient characteristics and pre-hospital variables which contribute to candidate selection, not on location of arrest.

Extracorporeal life support is a highly invasive procedure requiring significant medical resources and multi-disciplinary cooperation and a well-coordinated hospital system. It is challenging to make a prompt decision to devolve considerable resources and implement ECPR on the basis of incomplete medical information in sudden cardiac arrest occurring out-of-hospital or shortly after arrival at the emergency department (ED). Identifying predictive indicators of survival and developing a ECPR survival prediction model allows for prompt assessment of the effectiveness of ECPR during CPR. Furthermore, the identification of prognostic factors can help to minimize futile ECPR attempts and guide decisions on maintaining or withdrawing extracorporeal cardiopulmonary support. However, there are few studies examining predictors of good outcome in ECPR.

The objective of this study was to determine key indicators for good outcome in patients with sudden cardiac arrest undergoing ECPR and develop a prediction model to predict survival to hospital discharge in these patients.

## Methods

### Design and setting

This study was a retrospective analysis based on a prospective cohort study conducted at the emergency department (ED) of Korea University Medical Center (KUMC), between May 2006 and June 2016. We analyzed the CPR registry, which comprised prospectively collected data on pre-hospital and in-hospital variables of patients with cardiac arrest received CPR.

### Data collection for CPR registry

A CPR coordinator prospectively collected data for the CPR registry according to the Utstein-style guidelines [18, 19]. The registry included the following information: demographic data, comorbidities, whether the arrest was witnessed, the incidence of suspected or confirmed trauma, presumed arrest time; presence of bystander CPR, first documented arrest rhythm by the emergency medical service (EMS) provider, any return of spontaneous circulation (ROSC), presence of ECPR, the presence of return of spontaneous heart beating (ROSB) after ECPR, presumed cause of arrest; the application of therapeutic hypothermia and the use of coronary angiography (CAG) or percutaneous coronary intervention (PCI), 24-hour survival, the presence of ROSC ≥ 20 min, hospital length of stay (LOS), survival to hospital discharge, Glasgow–Pittsburgh cerebral performance category (CPC) score at discharge, and the final diagnosis at discharge. The comorbidity score was calculated using the Charlson comorbidity index [20]. The duration of CPR was defined as the time interval from the first chest compression provided by healthcare providers to the termination of resuscitation efforts due to ROSC (≥20 min), ROSB after ECPR, or a declaration of death. A favorable neurologic outcome was defined as a CPC score of 1 or 2 on the five-category scale.

### Indications and management of ECPR at the ED

The indications for ECPR at the KUMC-ED during the study period were as follows: 1) age ≥18 years, 2) sudden arrest with potentially reversible causes, 3) witnessed arrest with or without bystander CPR, or 4) a short no-flow time (time interval from presumed arrest to CPR initiation), even for unwitnessed arrests. The contraindications for ECPR were as follows: arrest due to a clearly uncorrectable cause, presence of a terminal illness or malignancy, severe irreversible neurologic deficit, suspected or confirmed traumatic origin of arrest, and no informed consent from the family.

The ECPR team was activated by the emergency physician in cases when cardiac arrest patients met the inclusion criteria, and required prolonged in-hospital CPR (>10 min) or suffered recurrent cardiac arrests in the ED after achievement of ROSC (≥20 min). The time from activating the ECPR team to implementation of ECPR was 10–15 min during the day and 20–25 min during the night.

The ECPR team consisted of emergency physicians, cardiovascular surgeons, coronary intervention specialists, and perfusionists. A twin pulse extracorporeal life support system (T-PLS^®^: NewHeartbio, Seoul, Korea), a Capiox emergency bypass system (EBS^®^; Terumo Inc., Tokyo, Japan), or a Permanent Life Support system (Maquet Cardiopulmonary GmbH, Rastatt, Germany) was used for ECPR. According to the patients’ body size, a 15- to 17-Fr arterial catheter and a 21- to 23-Fr venous catheter were inserted into the femoral artery and vein percutaneously by using Seldinger’s technique while maintaining chest compressions. The flow rate was initially set at 2.5–3.0 L/min. Anticoagulant with heparin was administered immediately after initiation of extracorporeal life support (ECLS) and titrated to maintain an activated clotting time of 200–220 s. After implementation of ECPR, CAG was performed as soon as possible in cases of suspected acute coronary syndrome.

Withdrawal of ECPR was considered if there was evidence of multiple organ failure, refractory shock, or irreversible neurologic injury and with consent from the patient’s family. A weaning protocol was instituted after assessing hemodynamic profiles and myocardial function by echocardiography while progressively reducing extracorporeal membrane oxygenation (ECMO) flow of 1.5 L/min [15].

### Study population and outcome

**Fig. 1:**
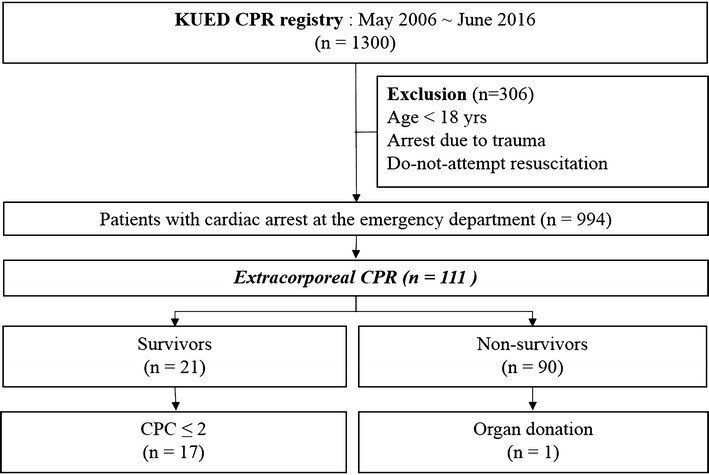
Flowchart of selection of study patients and outcomes

We enrolled adult patients (age ≥18 years) from the CPR registry cohort who underwent ECPR for OHCA or cardiac arrest shortly after arrival at the ED (Fig. 1). All cardiac arrest patients at the ED received advanced cardiac life support by emergency physicians according to the American Heart Association guidelines, excluding patients with a do-not-resuscitate order or irreversible signs of death.

The primary endpoint was a survival to discharge. We selected pre-ECPR variables with high statistical power for the prediction of survival at discharge.

### Data analysis

Values are presented as mean ± SD, median (interquartile ranges [IQRs]) for continuous variables, or number (%) of subjects for categorical variables.

Comparisons of baseline characteristics, CPR-related parameters, and post-resuscitation care variables between survivors and non-survivors were made using the Pearson *x* ^2^ test, Fisher’s exact test, Mann–Whitney *U* test, or Student’s *t* test according to the type of variable (Tables 1 and 2).

**Table 1:** Patient characteristics and cardiopulmonary resuscitation-related parameters

	Total (*n* = 111)	Survivors (*n* = 21)	Non-survivors (*n* = 90)	*p* value
Age (years)	55.9 ± 15.2	47.0 ± 14.8	57.9 ± 14.6	0.003
≤56, *n* (%)	53 (47.7)	16 (76.2)	37 (41.1)	0.004
>56, *n* (%)	58 (52.3)	5 (23.8)	53 (58.9)	
Male, *n* (%)	79 (71.2)	17(80.9)	62 (68.9)	0.272
Location of arrest				0.777
Out of hospital, *n* (%)	82 (73.9)	15 (71.4)	67 (74.4)	
Emergency department, *n* (%)	29 (26.1)	6 (23.6)	23 (25.6)	
Witnessed arrest, *n* (%)	95 (85.6)	20 (95.2)	75 (83.3)	0.298
Bystander CPR, *n* (%)	85 (76.6)	19 (90.5)	66 (73.3)	0.151
First documented rhythm				0.055
VF/VT, *n* (%)	53 (47.7)	14 (66.7)	39 (43.3)	
PEA, *n* (%)	32 (28.8)	6 (28.6)	26 (28.9)	
Asystole, *n* (%)	26 (23.4)	1 (4.8)	25 (27.8)	
Charlson comorbidity score <2	92 (82.9)	18 (85.7)	74 (82.2)	>.999
Presumed etiology of arrest				>.999
Cardiac, *n* (%)	104 (93.7)	20 (95.2)	84 (93.3)	
Non-cardiac, *n* (%)	7 (6.3)	1 (4.8)	6 (6.7)	
Time interval from arrest to CPR start by healthcare provider		3 (0–9)	4 (0–8)	0.987
CPR duration, min	56 (37–81)	51(34–55)	61(42–89)	0.022
≤55	53 (47.7)	16 (76.2)	37 (41.1)	0.004
>55	58 (52.3)	5 (23.8)	53 (58.9)	
Any ROSC event before ECPR, *n* (%)	39 (35.1)	11 (52.4)	28 (31.1)	0.066
Re-arrest after attaining ROSC ≥ 20 min, *n* (%)	26 (23.4)	7 (33.3)	19 (21.1)	0.258

Continuous variables are presented as mean ± SD or median (interquartile ranges). Categorical variables are presented as the number (%) of subjects

*CPR* Cardiopulmonary resuscitation, *VF*/*VT* ventricular fibrillation/pulseless ventricular fibrillation, *PEA* pulseless electrical activity, *ROSC* return of spontaneous circulation, *ECPR* extracorporeal cardiopulmonary resuscitation

**Table 2:** Post-resuscitation care and outcomes

	Total (*n* = 111)	Survivors (*n* = 21)	Non-survivors (*n* = 90)	*p* value
Initial laboratory data on admission to ED				
Serum hemoglobin, g/dL	13.1 ± 2.6	15.2 ± 2.2	12.6 ± 2.4	<0.001
Platelets (10^3^/µL)	181.6 ± 85.5	188.6 ± 91.3	180.1 ± 84.6	0.690
Serum lactate^a^, mmol/L	12.4 ± 5.3	12.6 ± 4.7	12.4 ± 5.4	0.870
Arterial pH^b^	7.04 ± 0.20	7.08 ± 0.23	7.03 ± 0.20	0.309
Serum bicarbonate^b^, mmol/L	16.1 ± 6.1	14.3 ± 5.2	16.5 ± 6.2	0.146
Base excess^b^, mmol/L	−14.6 ± 6.9	−15.3 ± 7.0	−14.4 ± 7.0	0.636
Blood urea nitrogen, mg/dL	19.6 ± 19.1	16.9 ± 6.6	20.2 ± 20.9	0.493
Serum creatinine, mg/dL	1.6 ± 2.0	1.3 ± 0.3	1.7 ± 2.2	0.495
AST (IU/L)	211 ± 551	208 ± 221	211 ± 603	0.982
ALT (IU/L)	168 ± 509	187 ± 224	163 ± 555	0.847
Total bilirubin, mg/dL	0.6 ± 0.5	0.6 ± 0.3	0.6 ± 0.5	0.760
aPTT (sec)	58.5 ± 45.0	52.1 ± 45.5	60.0 ± 45.0	0.484
PT (INR)	1.63 ± 1.99	1.25 ± 0.46	1.72 ± 2.19	0.340
SAPS II score^b^	92 ± 7	88 ± 6	93 ± 6	0.004
MAP ≥ 60 mmHg, after ECPR	62 (55.9)	19 (90.5)	43 (47.8)	<.001
Subsequent intervention after ECPR, *n* (%)				
Coronary angiography	89 (80.1)	19 (90.5)	70 (77.8)	0.238
Percutaneous coronary intervention	59 (53.2)	13 (61.9)	46 (51.1)	0.372
Therapeutic hypothermia, *n* (%)	31 (27.9)	9 (42.9)	22 (24.4)	0.09
24-h survival, *n* (%)	63 (56.8)	21 (100)	42 (46.7)	0.002
Time from arrest to ECPR, min	61 (42–88)	56 (34–66)	67(44–91)	0.042
ECLS duration, h	28 (6–58)	75 (42–124)	19 (4–48)	<.001
Hospital length of stay, days	1 (0–6)	30 (15–54)	0 (0–2)	<.001
Cerebral performance category				<.001
1 or 2	17 (15.3)	17 (81.0)	0	
3 or 4	5 (4.5)	4 (19.0)	1 (1.1)	
5 (organ donation)	1 (0.9)	0	1 (1.1)	

Continuous variables are presented as mean ± SD or median (interquartile ranges). Categorical variables are presented as the number (%) of subjects

ED, emergency department; ECPR, extracorporeal cardiopulmonary resuscitation; MAP, mean arterial blood pressure; ECLS, extracorporeal life support

^a^ Measured in 12 survivors and 75 non-survivors

^b^ Measured in 19 survivors and 85 non-survivors

In multivariable logistic regression, variables with *p* values <0.1 were chosen as candidate predictors and were entered into a logistic regression model (Table 1). Selected predictors (*p* ≤ 0.05) were age, CPR duration, first documented arrest rhythm, and any ROSC event before ECPR.

The optimal cutoff point of each relevant continuous predictor was assessed by the area under curve (AUC) in receiver operating characteristic (ROC) curve analysis. We then established a scoring system based on the predictors associated with survival to discharge outcome, assigning the weights according to logistic regression *β* coefficients. The model was retested for internal validation using bootstrap, with 1000 bootstrap replicates. We evaluated the discrimination using the AUC of the ROC curve. An AUC > 0.80 was considered to be an acceptable value. Model calibration was assessed using the Hosmer–Lemeshow goodness-of-fit test.

Analyses were performed using R-project version 3.2.2 (package “rms” version 5.1) and SPSS version 22.0 (IBM Corp, Armonk, NY). Two-tailed *p* values of <0.05 were considered to be statistically significant.

## Results

### Patient characteristics and CPR-related parameters

A total of 1300 patients with cardiac arrest at the ED were registered in the CPR registry during the study period. In all, 84.7% (*n* = 1100) of patients were covered by the public EMS system and 8.5% (*n* = 110) of patients were transferred using a private ambulance. A total of 78.8% (*n* = 1024) of patients were OHCA cases and 21.2% (*n* = 276) suffered cardiac arrest at the emergency department shortly after arrival.

Of the 1300 total patients in the registry, the 111 patients who underwent ECPR were enrolled in this study. There were 21 survivors and 90 non-survivors (Fig. 1). A comparison of characteristics and CPR-related variables is given in Table 1.

Eighty-two patients (73.9%) suffered OHCA and 104 patients (93.7%) had presumed cardiac etiologies. There was no difference in the location of arrest, etiology of arrest, witnessed arrest, and bystander CPR performed between survivors and non-survivors.

The survivors were younger and had a shorter CPR duration than the non-survivors (*p* = 0.003 and *p* = 0.022, respectively). The median CPR duration was 51 min (IQR 34–55 min) in survivors and 61 min (IQR 42–89 min) in non-survivors. In all, 66.7% of survivors had a shockable rhythm as the first documented rhythm, 52.4% of survivors had an event with any ROSC during CPR, and 33.3% of survivors experienced re-arrest following survival event (ROSC ≥ 20 min) (Table 1).

### Post-resuscitation care and outcomes

The initial serum hemoglobin on ED admission was higher among survivors (*p* < 0.001). Initial arterial pH, serum bicarbonate, renal function, liver function, and coagulation values did not differ between survivors and non-survivors. Pre-ECMO Simplified Acute Physiology Score (SAPS) II scores on admission were lower for survivors than for non-survivors using available data. There were no differences between the groups for subsequent interventions.

90.5% of survivors showed higher rate of mean arterial pressure (MAP) ≥60 mmHg after ECPR. A total of 90.5% of survivors had high mean arterial pressure (≥60 mmHg) after ECPR. In all, 61.9% of survivors received percutaneous coronary intervention due to occlusive lesions of the coronary artery and 9.5% (*n* = 2) of survivors were diagnosed with arrhythmia. Of the non-survivors, 5.6% (*n* = 5) had more than two extensive occlusive lesions. The median duration for maintaining ECLS was 75 h (IQR 42–124 h) in survivors and 19 h (IQR 4–48 h) in non-survivors. Favorable neurologic outcomes at discharge were achieved for 81% of survivors. One non-survivor became an organ donor. Most of non-survivors died within 3 days of the arrest (Table 2).

### Predictive indicators and prediction model for survival

Age, CPR duration, first documented rhythm (ventricular fibrillation [VF], ventricular tachycardia [VT], or pulseless electrical activity [PEA] vs. asystole), and any ROSC event before ECPR were significant indicators for survival:

$${\mathbf{log}}\left( {{\mathbf{p/}}\left( {{\mathbf{1}} - {\mathbf{p}}} \right)} \right) = 1.402 - 0.076 \times \left( {{\text{age}},\,{\text{years}}} \right) - 0.033 \times \left( {{\text{CPR}}\,{\text{duration}},\,{\text{hours}}} \right) + 1.754 \times \left( {{\text{any}}\,{\text{ROSC}}\,{\text{event}}} \right) + 2.490 \times \left( {{\text{first}}\,{\text{documented}}\,{\text{rhythm}}} \right)$$

**Fig. 2:**
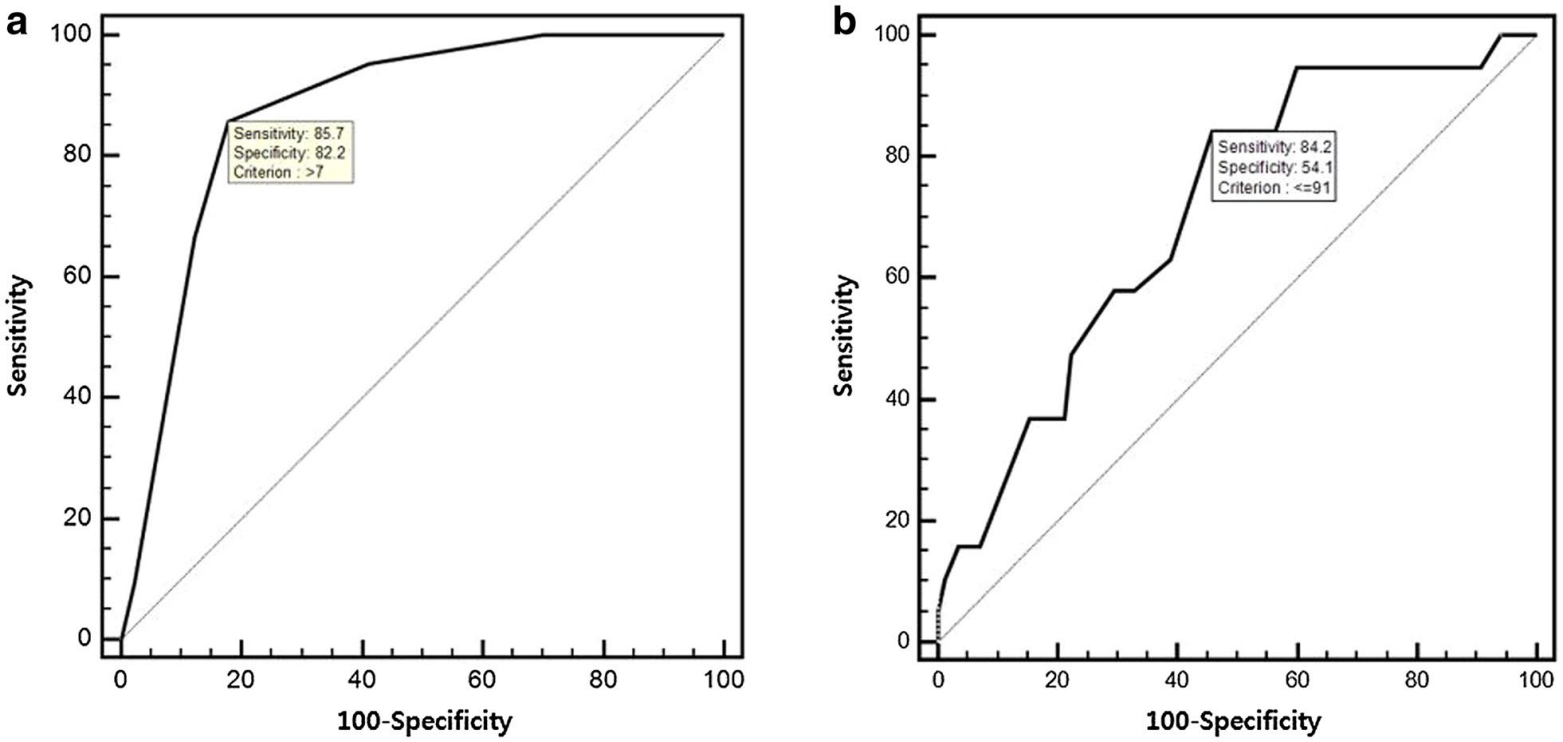
Receiver operating characteristic curves for predicting survival. **a** The prediction scoring model. **b** SAPS II

The optimal cutoff points for survival to discharge were as follows: age ≤56 years and CPR duration ≤55 min. Four indicators for predicting survival at discharge in patients undergoing ECPR were identified, and scores were assigned as follows: 3 points for age ≤56 years (odds ratio [OR] 7.57, 95% confidence interval [CI] 2.04–28.16), 4 points for CPR duration ≤55 min (OR 13.73, 95% CI 3.04–62.03), 3 points for VF/VT or PEA as first documented arrest rhythm (OR 8.3, 95% CI 0.98–70.64), and 3 points for any ROSC event before ECPR (OR 8.3, 1.97–34.98; Table 3). The prediction model for survival to discharge showed a c-statistic of 0.875 (95% CI 0.798–0.930, *p* < 0.001). The score-based prediction rule was developed from logistic regression equations using a regression coefficient-based scoring method. In the prediction scoring model, the c-statistic from internal validation using the bootstrapping method (number of repetitions = 1000) was comparable at 0.862 (95% CI 0.795–0.930). The cutoff value for the total score for survival was >7 points (sensitivity 85.7%, specificity 82.2%) (Fig. 2a). The SAPS II scoring system had a c-statistic of 0.707 (95% CI 0.609–0.792, *p* < 0.005) with a cutoff value of 91 points (Fig. 2b).

**Table 3:** Multivariate regression analysis of prognostic factors for survival to hospital discharge

	*β* coefficient	Odds ratio	95% CI	*p* value	Score
Age ≤ 56 years	2.02	7.57	2.04–28.16	0.003	3
CPR duration ≤ 55 min	2.62	13.73	3.04–62.03	<0.001	4
First documented arrest rhythm					
Asystole	0	1			
VF/pulseless VT and PEA	2.12	8.30	0.98–70.64	0.05	3
Any ROSC event before ECPR	2.12	8.3	1.97–34.98	0.004	3

*CI* Confidence interval, *CPR* cardiopulmonary resuscitation, *VF*/*VT* ventricular fibrillation/pulseless ventricular fibrillation, *PEA* pulseless electrical activity, *ROSC* return of spontaneous circulation, *ECPR* extracorporeal cardiopulmonary resuscitation

## Discussion

In this study, the key indicators for the survival of patients with ECPR were as follows: younger age (≤56 years), CPR duration up to 55 min, first documented cardiac rhythm without asystole, and any ROSC event during CPR. Our prediction model for survival after ECPR in the ED used these prognostic factors.

Several other studies reported prediction models for survival in veno-arterial extracorporeal membrane oxygenation (VA-ECMO) [21–24]. The survival after VA-ECMO (SAVE) model comprises 12 pre-ECMO variables and was developed using data from a large international cohort with a validation process. Parameters such as age, weight, cause of cardiogenic shock (diagnosis), chronic renal failure, acute pre-ECMO organ failure, peak inspiratory pressure, duration of mechanical ventilation, pulse pressure and diastolic pressure before ECMO, and serum bicarbonate value before ECMO were used in this model [21].

The SAVE scoring system can be useful for patients with available pre-VA-ECMO parameters in the intensive care unit during hospitalization; however, most pre-ECMO parameters are not available for patients with OHCA and IHCA at the ED, as in our study.

Peter et al. reported an association between Sequential Organ Failure Assessment (SOFA) and survival in VA-ECMO; however, the calculation of this score requires laboratory data, as well as the type and dose of inotropic agents [23]. Pre-ECMO SAPS II was also reported to be a predictor of mortality in VA-ECMO; however, vital signs, laboratory data (including serum bicarbonate), and history of chronic disease are necessary for the calculation of SAPS II [24]. SAPS II and SOFA scores are used to predict mortality in the setting of critical illness, such as in the intensive care unit, and are not specific models for patients with ECMO. Most parameters for these scores are not available or cannot be accessed for patients with OHCA or IHCA shortly after admission to the ED.

Studies on SAVE, SAPS II, and SOFA have included patients who need VA-ECMO support for cardiogenic shock, not patients who need VA-ECMO for cardiac arrest [22–24]. Even though the population in these studies included patients with cardiac arrest, those patients received ECMO support after IHCA, not OHCA. In our study, the prediction model for VA-ECMO survival focused on patients who received only ECPR at the ED. In all, 73.9% of the study population consisted of patients with OHCA. SAPS II may be a useful predictor in this study; however, the AUC of our model was higher than the AUC of the SAPS II scoring system. Moreover, the cutoff value of SAPS II was 91 points for ECPR, which is higher than the 80-point cutoff value for VA-ECMO in the study by Lee et al. [24]. Our prediction model may be useful for selecting and treating patients who need ECMO support during CPR but do not have patient information and laboratory data available at the ED.

The optimal cutoff age was 56 years for patients with sudden cardiac arrest at the ED or OHCA; there were no survivors older than 75 years. Other studies of prediction models for survival after ECPR for in-hospital cardiac arrest have identified a cutoff age of 66 years [16, 25]. This discrepancy may be due to the different patient characteristics between ED cardiac arrests and IHCAs, such as younger age, incomplete medical information, and pre-hospital variables for ECPR at the ED.

Some studies have reported that an age of <75 years predicts survival in CCPR [26, 27]. Maupain et al. also reported that old age was a risk factor for poor neurologic outcomes after CCPR for OHCA [28]. As the upper age limit for ECPR is generally set at 75 years, clinical data including very elderly patients with ECPR are rare [2, 14]. Younger patients with refractory cardiac arrest are good candidates for ECPR; however, the upper age limit for ECPR has not been validated [15].

The optimal time for implementation of ECPR, according to CPR duration, has been reported as follows: <30–60 min for survival [29, 30] and <55.5–80 min for favorable good neurologic outcomes [15, 31, 32]. A CPR duration of less than 55 min was an indicator of high likelihood of survival in our study. A meta-analysis by Debaty et al. [33] also concluded that shorter CPR duration was a prognostic factor in ECPR, associated with survival.

Reynolds et al. reported that the rate of favorable neurologic outcomes in OHCA decreased after 16 min of CPR and adequate CPR duration for probability of favorable neurologic outcome can be dynamically prolonged according to CPR-related variables, such as shockable arrest rhythm, witnessed arrest, and bystander CPR [10, 34]. As ECPR provides sufficient perfusion to vital organs, the window for an effective resuscitation duration can be extended [30, 31]. CPR duration is an indicator for the implementation of ECPR as well as an index to explain refractoriness to CCPR [35]. Providing ECPR to patients with refractory arrest within an optimal CPR duration is critical to achieve favorable outcomes.

The initial arrest rhythm (VF/pulseless VT or PEA, but not asystole) was found to be a predictive indicator of survival for ECPR patients. Other studies have indicated that a shockable arrest rhythm and witnessed arrest are prognostic indicators of survival and favorable neurologic outcome following CCPR [27, 28, 34, 36]. A shockable rhythm can be interpreted as a brief no-flow time or as an arrest of presumed cardiac etiology, which has also been reported to be a major factor of good outcomes in ECPR [12, 14, 30, 33]. Furthermore, studies by Park et al. and Kim et al. found that PEA was associated with survival and favorable neurologic outcomes in ECPR [15, 37]. When analyzed using three-categorized arrest rhythm, the prognostic factors for survival were as follows: age ≤56 years (OR 7.66, 95% CI 2.00–29.25, *p* = 0.003), CPR duration ≤55 min (OR 13.97, 95% CI 3.11–62.74, *p* < 0.001), VF/VT (OR 10.24, 95% CI 1.52–90.98, *p* = 0.037), PEA (OR 5.56, 95% CI 0.55–56.23 *p* = 0.146), and any ROSC event before ECPR (OR 8.3, 2.06–35.03, *p* = 0.003). The *β* coefficient of PEA was 1.72, whereas the *β* coefficient of shockable rhythm was 2.33. PEA was not a strong prognostic factor such as VF/pulseless VT, but one of predictors. The likelihood of a reversible etiology of arrest needs to be considered in the patients with PEA. ECLS plays a role as a bridge from refractory arrest to the correction of reversible causes of the arrest by ensuring adequate delivery of oxygenated blood until effective cardiac output has been restored. ECPR can be considered in the patients with PEA. However, implementation of ECPR for the patients with asystole is not recommended.

We found no differences between survivors and non-survivors with regard to pre-hospital variables, such as witnessed arrest and bystander CPR, in our univariate analysis. Witnessed arrest has been shown to be a prognostic factor for favorable outcomes in CCPR [26, 34]. However, this has not been clearly demonstrated for ECPR [12, 33, 38]. In this study, as in previous studies, prerequisites for ECPR included witnessed cardiac arrest or brief no-flow time; therefore, no association between witnessed arrest and survival could be identified.

The prediction model identified a ROSC event before ECPR as a predictor of survival in ECPR. CCPR achieves 25% of cardiac output, and high-quality CPR is important to achieve the necessary perfusion to major organs by compression [1, 35]. Any ROSC event can decrease low-flow duration with minimal cardiac output and increase the duration of perfusion.

In our univariate analysis, there were more patients without preexisting comorbidities in the survival group than in the non-survival group. The application of ECPR was usually limited to patients with a low Carlson comorbidity index (0 or 1). No differences in types or burden of comorbidities were detected [15].

The association between comorbidities and survival is not clear in ECPR, although Sǿholm et al. [39] found that comorbidity (low Charlson comorbidity index ≤2) was not independently associated with outcomes in CCPR. Similarly, comorbidity was not a prognostic factor in our study.

There were no differences between survivors and non-survivors with regard to in-hospital variables, such as serum lactate levels, and arterial pH as markers of inadequate tissue oxygenation and perfusion on ED admission. Lower lactate levels and higher arterial pH values have been reported among ECPR survivors [29, 33]. Serum hemoglobin levels on admission were associated with survival in this study. Hemoglobin levels can decrease due to outflow to the ECMO circuit and inflow from crystalloid solution of ECMO priming after ECPR. As decreased hemoglobin levels are associated with decreased oxygen delivery, impaired tissue function improvements in ischemic events during cardiac arrest may depend on the hemoglobin level [40, 41]. A mean arterial pressure ≥60 mmHg after ECPR was a dependent factor through hemodynamic optimization.

## Limitations

This study has several limitations that require consideration. First, this was a non-randomized observational cohort study using retrospective analysis in a single center.

Only a small number of patients from a single medical center were included in this study, so the scoring system had limited power. Moreover, external validation of the scoring system was not performed due to the insufficient number of cases. The small cohort prevented many parameters from achieving statistical significance and restricted detailed analysis. Additionally, it limits the application of our results in other hospitals. A large multicenter study is needed for external validation and comparison with other scoring systems.

Second, the indications for ECPR in selected patients were limited and only small number of patients was included. ECPR may improve survival and neurologic outcomes post-arrest in limited and carefully selected cases. However, the use of ECPR remains controversial and other studies have applied different criteria [12, 14]. The criteria for the implementation of ECPR have not been established, with indications and protocols differing according to regional variations of EMS and in-hospital systems [12]. In this study, predefined inclusion criteria (witnessed arrest or presumed brief no-flow time) were applied to select patients for ECPR. Patients with sudden cardiac arrest are generally younger, have fewer chronic comorbidities, and are more likely to suffer a sudden arrest of cardiac origin, particularly in OHCA [16, 25]. The inclusion criteria and the specific characteristics of patients with sudden cardiac arrest in the ED are different from those of patients with IHCA and must be considered when interpreting prognostic factors. As indications for ECPR in this study included witnessed arrest or arrest with brief no-flow time, the prognostic effect of witnessed arrests may have been overlooked.

Third, ECLS outcomes may be dependent on patient characteristics, pre-hospital CPR variables (including witnessed arrest), bystander CPR, a multifaceted approach to treat reversible causes of arrest, EMS systems, and ECMO team expertise [42]. Berdowski et al. reported that OHCA incidence reported that OHCA incidence and outcomes vary, with the rates of VF as the cardiac arrest rhythm as follows: approximately 31.6% in Europe, 30.4% in North America, 39.0% in Australia, and 7.4% in Asia. Such global and regional variabilities need to be considered for analysis [43]. Moreover, differences in the availability and quality of ECPR teams, hospital facilities, infrastructure, and pre-hospital emergency response systems, according to regional and national variations can lead to different outcomes.

Finally, we were unable to determine the statistical significance of laboratory parameters due to the small number of ECPR cases in our study. Furthermore, 87.8% of non-survivors died within 3 days of admission. Because most of these deaths occurred in the acute period, many laboratory parameters could not be obtained. Therefore, the effects of these unavailable and missing data on the scoring cannot be fully assessed.

## Conclusions

In our study, younger age, shorter CPR duration, any ROSC event before ECPR, and no asystole as the first documented arrest rhythm were predictive indicators for survival in patients who received ECPR. With careful consideration of differences in the inclusion criteria, the prognostic indicators and prediction scoring model in our study may be helpful in the rapid decision-making process for ECPR implementation during CPR in the ED. However, because this was a small-sized observational study, a future multicenter cohort-based study is needed.

## Abbreviations

AUC: area under the curve
CCPR: conventional cardiopulmonary resuscitation
CI: confidence interval
CPR: cardiopulmonary resuscitation
ECLS: extracorporeal life support
ECMO: extracorporeal membrane oxygenation
ECPR: extracorporeal cardiopulmonary resuscitation
ED: emergency department
EMS: emergency medical services
IHCA: in-hospital cardiac arrest
IQR: interquartile range
KUMC: Korea University Medical Center
OHCA: out-of-hospital cardiac arrest
OR: odds ratio
PEA: pulseless electrical activity
ROC: receiver operating characteristic
ROSB: return of spontaneous heart beating
ROSC: return of spontaneous circulation
SAPS: Simplified Acute Physiology Score
SAVE: survival after veno-arterial extracorporeal membrane oxygenation
SOFA: Sequential Organ Failure Assessment
VA: venoarterial
VF: Ventricular fibrillation
VT: ventricular tachycardia
